# Treatment of Diphtheria, Inflammatory Angina and Croup, by the Perchloride of Iron

**Published:** 1861-02

**Authors:** M. Aubran


*Treatment of Diphtheria, Inaflmmatory Angina and Croup, by the Perchloride of Iron, by* M. AUBRAN.—The author read a paper with the above title at the meeting of the Academy of Sciences, December 7th. The following is his plan of treatment: "I put from twenty to forty drops of perchloride of iron into a glass of cold water, according to the gravity of the disease and the age of the patient. The patient must drink a swallow (about two dessert spoonfuls) every five minutes while he is awake, and every quarter of an hour during sleep. Immediately after each dose, a swallow of cold milk without sugar should be given. This treatment must be continued with regularity for several days, without respect to sleep for the first three days. Experience has taught me that it is only at the end of the third day that false membranes begin to soften and be detached. This solution of perchloride of iron ought always to be administered in a glass or porcelain vessel, so as to avoid the decomposition which takes place when it comes in contact with metal. I forbid all drinks and nourishment susceptible of decomposing the iron. In general, during the first three or four days, I do not give anything but the solution of perchloride of iron and cold milk. Local treatment is secondary, and may even be entirely omitted. The internal treatment is sufficient in the greatest number of cases. Administered from the beginning of the disease, this medication will cure the oftenest without an operation. If the progress of the croup is very rapid, or if the medication has not been given until an advanced period of the disease, tracheotomy may become necessary, but the perchloride of iron should be continued, for it is it alone which will cure. Of thirty-nine cases, during the first three days thirty-five have been cured; two cases only have required tracheotomy, from the beginning of the treatment. It was continued scrupulously, and a cure resulted in the two cases, in spite of the gravity of the disease, as the dipetheritic membrane had invaded the bronchia to a great extent."—*Cin. Lan. & Observer.*

